# Supplementary material for: The effects of genital myiasis on the diversity of the vaginal microbiota in female Bactrian camels
Source: BMC Vet Res. 2022 Mar 5;18:87. doi: 10.1186/s12917-022-03189-5 (PMC8897907; doi:10.1186/s12917-022-03189-5)
Supplement: Supplementary file 5 — Additional file 5. [file 12917_2022_3189_MOESM5_ESM.zip › MPL201709200_16s_yy/Treat1/css/js_css/pdfmake-master/examples/pdfs/margins.pdf]

This header has both top and bottom margins defined

This is a subheader

Margins have slightly different behavior than other layout properties. They are not inherited, unlike anything else. They're applied only to those nodes which explicitly set margin or style property.

This paragraph (consisting of a single line) directly sets top and bottom margin to 20

This line begins a stack of paragraphs. The whole stack uses a *superMargin* style (with margin and fontSize properties).

When you look at the *document definition*, you will notice that fontSize is inherited by all paragraphs inside the stack.

Margin however is only applied once (to the whole stack).

I'm not sure yet if this is the desired behavior. I find it a better approach however. One thing to be considered in the future is an explicit layout property called inheritMargin which could opt-in the inheritance.

Currently margins for inlines are ignored

Lorem ipsum dolor sit amet, consectetur adipisicing elit. Malit profecta versatur nomine ocurreret multavit, officiis viveremus aeternum superstitio suspicor alia nostram, quando nostros congressus susceperant concederetur leguntur iam, vigiliae democritea tantopere causae, atilii plerumque ipsas potitur pertineant multis rem quaeri pro, legendum didicisse credere ex maluisset per videtis. Cur discordans praetereat aliae ruinae dirigentur orestem eodem, praetermittenda divinum. Collegisti, deteriora malint loquuntur officii cotidie finitas referri doleamus ambigua acute. Adhaesiones ratione beate arbitraretur detractis perdiscere,

constituant hostis polyaeno. Diu concederetur.
Lorem ipsum dolor sit amet, consectetur adipisicing elit. Malit profecta versatur nomine ocurreret multavit, officiis viveremus aeternum superstitio suspicor alia nostram, quando nostros congressus susceperant concederetur leguntur iam, vigiliae democritea tantopere causae, atilii plerumque ipsas potitur pertineant multis rem quaeri pro, legendum didicisse credere ex maluisset per videtis. Cur discordans praetereat aliae ruinae dirigentur orestem eodem, praetermittenda divinum. Collegisti, deteriora malint loquuntur officii cotidie finitas referri doleamus ambigua acute. Adhaesiones ratione beate arbitraretur detractis perdiscere, constituant hostis polyaeno. Diu concederetur.
Lorem ipsum dolor sit amet, consectetur adipisicing elit. Malit profecta versatur nomine ocurreret multavit, officiis viveremus aeternum superstitio suspicor alia nostram, quando nostros congressus susceperant concederetur leguntur iam, vigiliae democritea tantopere causae, atilii plerumque ipsas potitur pertineant multis rem quaeri pro, legendum didicisse credere ex maluisset per videtis. Cur discordans praetereat aliae ruinae dirigentur orestem eodem, praetermittenda divinum. Collegisti, deteriora malint loquuntur officii cotidie finitas referri doleamus ambigua acute. Adhaesiones ratione beate arbitraretur detractis perdiscere, constituant hostis polyaeno. Diu concederetur.
Lorem ipsum dolor sit amet, consectetur adipisicing elit. Malit profecta versatur nomine ocurreret multavit, officiis viveremus aeternum superstitio suspicor alia nostram, quando nostros congressus susceperant concederetur leguntur iam, vigiliae democritea tantopere causae, atilii plerumque ipsas potitur pertineant multis rem quaeri pro, legendum didicisse credere ex maluisset per videtis. Cur discordans praetereat aliae ruinae dirigentur orestem eodem, praetermittenda divinum. Collegisti, deteriora malint loquuntur officii cotidie finitas referri doleamus ambigua acute. Adhaesiones ratione beate arbitraretur detractis perdiscere, constituant hostis polyaeno. Diu concederetur.
Lorem ipsum dolor sit amet, consectetur adipisicing elit. Malit profecta versatur nomine ocurreret multavit, officiis viveremus aeternum superstitio suspicor alia nostram, quando nostros congressus susceperant concederetur leguntur iam, vigiliae democritea tantopere causae, atilii plerumque ipsas potitur pertineant multis rem quaeri pro, legendum didicisse credere ex maluisset per videtis. Cur discordans praetereat aliae ruinae dirigentur orestem eodem, praetermittenda divinum. Collegisti, deteriora malint loquuntur officii cotidie finitas referri doleamus ambigua acute. Adhaesiones ratione beate arbitraretur detractis perdiscere, constituant hostis polyaeno. Diu concederetur.
Lorem ipsum dolor sit amet, consectetur adipisicing elit. Malit profecta versatur nomine ocurreret multavit, officiis viveremus aeternum superstitio suspicor alia nostram, quando nostros congressus susceperant concederetur leguntur iam, vigiliae democritea tantopere causae, atilii plerumque ipsas potitur pertineant multis rem quaeri pro, legendum didicisse credere ex maluisset per videtis. Cur discordans praetereat aliae ruinae dirigentur orestem eodem, praetermittenda divinum. Collegisti, deteriora malint loquuntur officii cotidie finitas referri doleamus ambigua acute. Adhaesiones ratione beate arbitraretur detractis perdiscere, constituant hostis polyaeno. Diu concederetur.
Lorem ipsum dolor sit amet, consectetur adipisicing elit. Malit profecta versatur nomine ocurreret multavit, officiis viveremus aeternum superstitio suspicor alia nostram, quando nostros congressus susceperant concederetur leguntur iam, vigiliae democritea tantopere causae, atilii plerumque ipsas potitur pertineant multis rem quaeri pro, legendum didicisse credere ex maluisset per videtis. Cur discordans praetereat aliae ruinae dirigentur orestem eodem, praetermittenda divinum. Collegisti, deteriora malint loquuntur officii cotidie finitas referri doleamus ambigua acute. Adhaesiones ratione beate arbitraretur detractis perdiscere,

constituant hostis polyaeno. Diu concederetur.
